# The loss of function of *HEL*, which encodes a cellulose synthase interactive protein, causes helical and vine-like growth of tomato

**DOI:** 10.1038/s41438-020-00402-0

**Published:** 2020-11-01

**Authors:** Qihong Yang, Xiaoshuai Wan, Jiaying Wang, Yuyang Zhang, Junhong Zhang, Taotao Wang, Changxian Yang, Zhibiao Ye

**Affiliations:** Key Laboratory of Horticultural Plant Biology, Ministry of Education, Huazhong Agricultural University, Wuhan, 430070 China

**Keywords:** Plant morphogenesis, Transgenic plants

## Abstract

Helical growth is an economical way for plant to obtain resources. The classic microtubule–microfibril alignment model of *Arabidopsis* helical growth involves restriction of the appropriate orientation of cellulose microfibrils appropriately in the cell walls. However, the molecular mechanism underlying tomato helical growth remains unknown. Here, we identified a spontaneous tomato *helical* (*hel*) mutant with right-handed helical cotyledons and petals but left-handed helical stems and true leaves. Genetic analysis revealed that the *hel* phenotype was controlled by a single recessive gene. Using map-based cloning, we cloned the *HEL* gene, which encodes a cellulose interacting protein homologous to CSI1 of *Arabidopsis*. We identified a 27 bp fragment replacement that generated a premature stop codon. Transgenic experiments showed that the helical growth phenotype could be restored by the allele of this gene from wild-type *Pyriforme*. In contrast, the knockout mutation of *HEL* in *Pyriforme* via CRISPR/Cas9 resulted in helical growth. These findings shed light on the molecular control of the helical growth of tomato.

## Introduction

The rational use of growing space is essential for plant photosynthesis and reproductive development and indirectly affects biotic and abiotic resistance. Stretched leaves, upright growth, and deep roots enable more solar radiation and CO_2_ to be absorbed, thereby minimizing intraplant competition, to achieve increased photosynthesis efficiency^1^. A growth axis is usually generated in plants due to the expansion of organs into space, and a vast majority of plants display linear or circumferential growth^2^. Notably, due to the lack of strong xylem support, some plants evolve a helical growth axis to achieve increased heights by winding onto a support, as observed for climbing plants. Specific organs of climbing plants, such as tendrils in *Vitis*, *Pisum sativum*, and *Cucurbitaceae*, often exhibit helical growth^3^. In addition, members of the legume family and other vine plants climb through stem circumnutation, the vast majority of which display a right-handed helix^4^. For some nonclimbing plants, pruning and bundling are essential for improving photosynthesis efficiency, and then yield and quality in agriculture.

Helical growth direction can be described as left- or right-handedness^4^. The left-handed helix involves clockwise rotation, as viewed from above. In contrast, right-handedness involves rotation in an anticlockwise direction. This phenomenon in climbing plants was described by Darwin a century ago. However, the molecular mechanisms underlying helical growth have been revealed only in the past two decades. Two loss-of-function mutations in *Arabidopsis*, *SPIRAL 1* (*SPR1*) and *SPIRAL 2* (*SPR2*), were first discovered to be responsible for right-handed helical growth^5^. Between them, the former caused right-handed helical growth of roots, stems, and hypocotyls, whereas the latter even extended to leaf petioles and petals. The *spr1/spr2* double mutant exhibited a more obvious phenotype than did each single mutant^5^. In addition, mutations in *TUA4* and *TUA6* which separately encode α-tubulin 4 and α-tubulin 6 resulted in the left-handed twisting of roots, cotyledons, leaves, and flowers^6^. Previous studies suggested that the cytoskeleton is closely related to helical growth of *Arabidopsis*^5^. Cortical microtubule (CMT) arrays have been shown to be oriented opposite to that of spiral growth of epidermal cell files^6^. SPR1, a microtubule-associated protein (MAP) that localizes to the plus-end of microtubules (MTs), can stabilize the growing ends of CMTs and influence their dynamic properties^7,8^. Another plant-specific MAP, SPR2, is involved in the modulation of MT severity and regulates both the orientation of CMTs and the direction of organ growth^9,10^. Moreover, a series of mutations in tubulins or MAPs led to left- or right-handed helical growth of *Arabidopsis*, suggesting that various genetic mutations can cause twisted-type growth of several directions^11–13^.

CMTs, which are organized in arrays parallel to the central axis of cell expansion, inside the plasma membrane play an important role in controlling cell wall status and determining final cell shape^14^. The elongation or expansion of plant cells is restricted by the cell wall. Cellulose microfibrils (CMFs), which are synthesized by the cellulose synthase complex (CSC), act as the main factors linking CMTs and the cell wall during anisotropic growth of *Arabidopsis*^15–18^. Moreover, cellulose synthase interactive protein 1 (CSI1) functions as an interacting factor between CSCs and CMTs^18–21^. Knockout of *Arabidopsis* CSI1 caused left-handed helical growth of nearly all elongating organs, except for inflorescence stems, which exhibited right-handed twisting^20,21^. CSI1 binds to both CSCs and CMTs during cell wall formation and acts as a tow cable involved in the movement of cellulose synthase rosettes^19,21^. Therefore, CSI1 constitutes a critical part of the microtubule–microfibril alignment model.

Mutational cytoskeletal-related genes in *Arabidopsis* provide cellular and genetic evidence that help us understand helical growth. The vegetative shoot apical meristem in *Arabidopsis* gives rise to leaves until inflorescence development^22^. The determinate growth habit of *Arabidopsis* is distinct from that of indeterminate plant species, such as tomato. Vegetative and reproductive phases of tomato regularly alternate, which results in an indeterminate plant^23^. Therefore, the suitability of microtubule-mediated guidance during cellulose microfibril pattern formation in indeterminate tomato cultivars is still unclear. As observed for this type of tomato cultivar, cultivation requires plant supports such as garden stakes and twines to keep the tomato plant in an upright state.

In this study, we identified the *HEL* gene, which encodes a cellulose synthase-interactive protein, via map-based cloning. Loss of function of the *HEL* gene caused right-handed helical growth of cotyledons and petals, whereas the opposite direction of stems and true leaves was observed in tomato. These results suggest that the function of *HEL* is critical for helical growth of tomato, which provides a foundation for understanding the helical architectures of tomato.

## Materials and methods

### Plant materials and mapping populations

We identified a spontaneous mutant that exhibits a helical growth phenotype in the background of *Pyriforme* (*Solanum lycopersicum* var. *cerasiforme*), which is hereafter referred to as the *hel* mutant. We developed an F_2_ segregating population by crossing the *hel* mutant with LA1589 (*Solanum pimpinellifolium*). The *hel* mutant and *Pyriforme* were used as recipients for genetic transformation. All materials were grown in a greenhouse under standard conditions for phenotypic analysis. The height of the *hel* mutants and *Pyriforme* plants was measured once a week for a month. Eighteen tomato fruits were collected from three plants (6 fruits per plant), and their weight and fruit shape index were measured. In addition, the fruit set was measured after fruit enlargement. Three biological replicates were included.

### Extraction of RNA for RNA-seq

Thirty wild-type and mutant F_2_ individuals were collected and separately pooled as the N-pool and M-pool, respectively, based on their phenotypes. Total RNA was extracted using TRIzol reagent (Invitrogen, USA). After treatment with RNase-free DNase I (Takara, Japan), the RNA quantity and quality were then tested by using a NanoDrop 2000 instrument (Thermo Scientific, USA). RNA-seq libraries were generated using a NEBNext Ultra II RNA Library Prep Kit for Illumina (New England Biolabs, USA), according to the manufacturer’s protocol. The libraries were subsequently sequenced on an Illumina HiSeq2000 platform (Novogene, China).

### BSR-seq mapping of *HEL*

Raw reads from the RNA-seq experiment were scanned for quality and trimmed to remove low-quality bases. The clean reads from the N-pool and M-pool were mapped to the reference genome (Heinz1706) using BWA software (Availability: http://bio-bwa.sourceforge.net)^24^. SNPs were further identified and quantified at each SNP site in both pools using SAM software (Availability: http://bio-bwa.sourceforge.net)^24^. BSR-seq analysis was performed to determine the chromosomal location of *HEL* according to the method described by Liu et al.^25^. Because both parents are homozygous lines, only homozygous SNPs were retained in both parents. The parameters of the SNP index were calculated to identify candidate regions for *HEL*. The average SNP index for a given genomic interval was estimated using a sliding window approach, with a 1 Mb window size and 10 kb increment. A SNP index graph was subsequently constructed and plotted.

### DNA extraction and molecular marker generation

Genomic DNA was isolated from young leaves of 4-week-old tomato seedlings according to the method described by Fulton et al.^26^. Based on the nucleotide polymorphism identified from the *hel* mutant and LA1589 in the target region, three types of molecular markers were developed: InDel, SNP, and CAPS markers. All the primers used are listed in Table S1.

### Genotyping of F_2_ individuals

Genotyping of F_2_ individuals was performed via PCR using polymorphic molecular markers. The PCR mixture consisted of 1 μL of DNA, 2 μL of 10× PCR buffer, 0.4 μL of dNTPs (10 mM), 0.2 μL of 2.5 U/μL Taq DNA polymerase (Invitrogen, USA), 0.4 μL of each primer (10 pm/μL), and 15.6 μL of distilled water. The reaction parameters are described as follows: initial denaturation at 94 °C for 3 min; 35 cycles at 94 °C for 30 s, 56 °C for 30 s, and 72 °C for 1 min; and a final extension step at 72 °C for 10 min. The PCR products were then analyzed with a 1.5% (w/v) agarose gel. For CAPS markers, the amplification products were further digested with appropriate restriction enzymes and then analyzed with a 1.5% (w/v) agarose gel. The PCR-based SNP markers were genotyped via Sanger sequencing.

### Genetic mapping

For all the polymorphic markers between the *hel* mutant and LA1589, 108 F_2_ recessive individuals were initially used for rough mapping of the *HEL* gene. According to the rough mapping of *HEL*, new molecular markers were developed. Recombinant events were within a population of 925 individuals further screened using newly developed markers. The Genotypic and phenotypic data of F_2_ recessive individuals were adopted for linkage analysis using the MapMaker/EXP 3.0 program^27^. The genetic distance was computed using the Kosambi mapping function.

### Gene prediction and sequence analysis

The candidate genes in the target region between two closely linked markers were downloaded from the SGN database (ftp://sgn.cornell.edu). These genes were further analyzed using FGENESH and GeneScan. The primers used were designed based on the sequence information of these candidates using Primer 5.0. We amplified and sequenced the genomic and cDNA sequences of these candidates from the *hel* mutant and *Pyriforme*. The sequences were aligned with ClustalW2 and edited with GeneDoc. All the primers used are listed in Supplementary Table S2.

### Vector construction and plant transformation

The full-length coding sequence of ORF2 was cloned into pHellsgate8 driven by the CaMV 35S promoter. For CRISPR/Cas9 vector construction, we selected two adjacent target sites separated by a 340 bp spacer in this candidate. These two constructs were separately transformed into *hel* mutants and *Pyriforme* via *Agrobacterium tumefaciens* strain GV3101^28^. The resulting transgenic plants were screened with gene-specific primers.

## Results

### Phenotypic analyses of the *hel* mutant

**Fig. 1:**
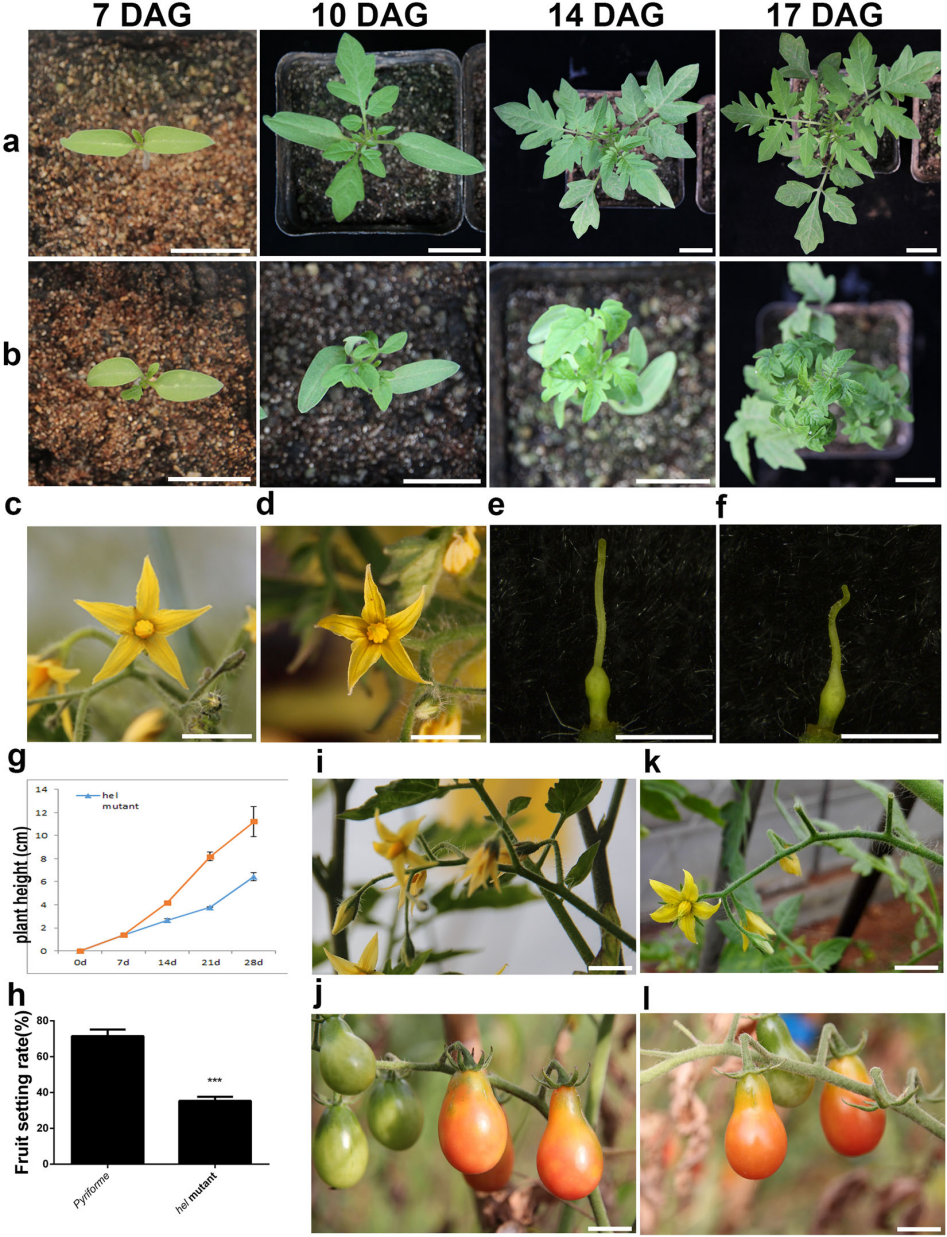
Phenotypic changes of the *hel* mutant. **a**, **b** Seedlings of *Pyriforme* and the *hel* mutant at 7, 10, 14, 17 DAG, respectively. The right-handed helical growth of leaves in the *hel* mutant shifted to left-handedness between the 3rd and 6th leaves (at approximately 14 DAG). DAG, days after germination. **c**, **d** Petals of *Pyriforme* and the *hel* mutant. **e,** **f** Pistils of *Pyriforme* and the *hel* mutant. **g** Plant height of *Pyriforme* and the *hel* mutant. The error bars are the standard deviations. **h** Fruit set of *Pyriforme* and the *hel* mutant. Significant differences are represented by black asterisks, ***P < 0.0001. **i**, **k** Inflorescence branch of *Pyriforme* and the *hel* mutant, respectively. **j**, **l** Fruits of *Pyriforme* and the *hel* mutant, respectively. Scale bars, 2 cm (**a**, **b**), 1 cm (**c**, **d**, **i**–**l**), 1 mm (**e**, **f**)

We visually examined the helical growth phenotypes of the *hel* mutant. We found that the cotyledons, petals and pistils of the *hel* mutant showed right-handed helical growth (Fig. 1a–f). Compared with wild type, the *hel* mutant seedlings showed a lagging growth momentum at an early stage (Fig. 1g and Supplementary Fig. S1a). Interestingly, the stems showed no helical growth at this stage. However, the growth direction of *hel* stems gradually shifted to left-handedness after flowering (Supplementary Fig. S1a–c). Similarly, the right-handed growth direction of the true leaves gradually shifted to left-handed twisting between the 3rd and 6th true leaves (Fig. 1a, b and Supplementary Fig. S1a). The *hel* phenotypes of the sepals, anther cones and inflorescence axis exhibited no obvious changes in comparison with those of the wild type (Fig. 1i–l and Supplementary Fig. S1d–g). Notably, the pistil length and fruit set of the *hel* mutant were significantly reduced (Fig. 1e, f, h). Moreover, sizes of the flowers and fruits showed no significant changes (Fig. 1c, d, j, l).

**Fig. 2:**
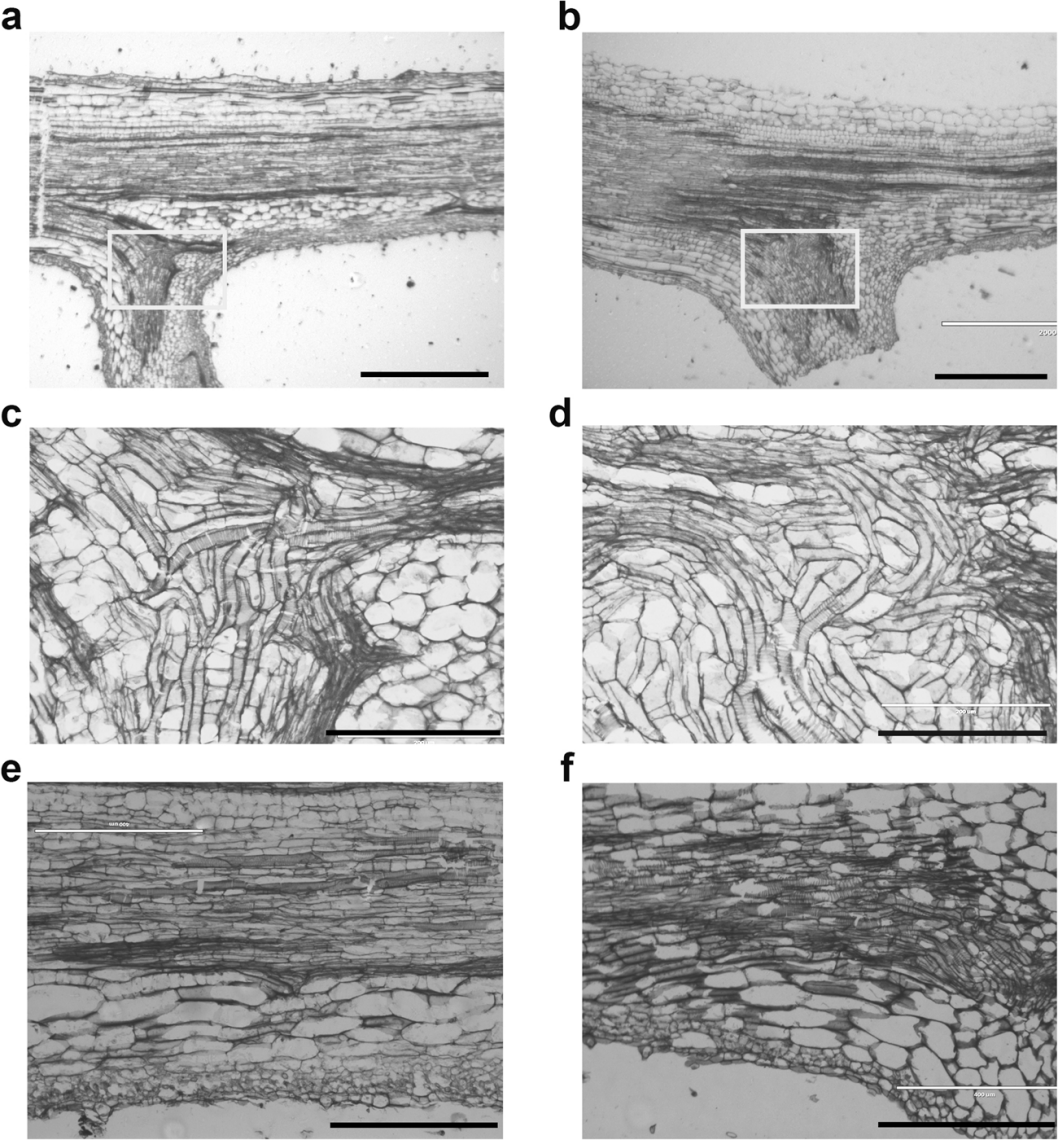
Cytological observations of the *hel* mutant and its *Pyriforme* wild-type background. **a**, **b** Transverse sections of the rachis of the first compound leaflet of *Pyriforme* and the *hel* mutant, respectively. The rectangle indicates the subsequent magnified area. **c**, **d** Transverse sections of the petiolules of *Pyriforme* and the *hel* mutant, respectively. **e**, **f** Transverse sections of the rachises of the wild type and *hel* mutant. Scale bars, 1000 μm (**a**, **b**), 200 μm (**c**, **d**), 400 μm (**e**, **f**)

To detect the possible cytological changes underlying the helical growth of the *hel* mutant, we conducted cytological observations of the petiolules and rachises of compound leaflets using the paraffin sectioning technique. We found that the basal cells of petiolules were vertically arranged in wild type (Fig. 2a, c) but twisted in the *hel* mutant (Fig. 2b, d). Moreover, the cell arrangement exhibited a parallel arrangement in the wild-type rachis, whereas it was distorted in the rachis of the *hel* mutants (Fig. 2e, f).

We generated an F_2_ segregating population from *hel* mutant×LA1589 cross. The F_2_ individuals comprised 344 wild-type and 108 helical plants, consistent with a 3:1 mendelian ratio (*χ*^2^ = 0.103, *P* > 0.05). In combination with the normal phenotype of F_1_ plants, we inferred that the helical phenotype was controlled by a recessive gene.

### Map-based cloning of the *HEL* gene

**Fig. 3:**
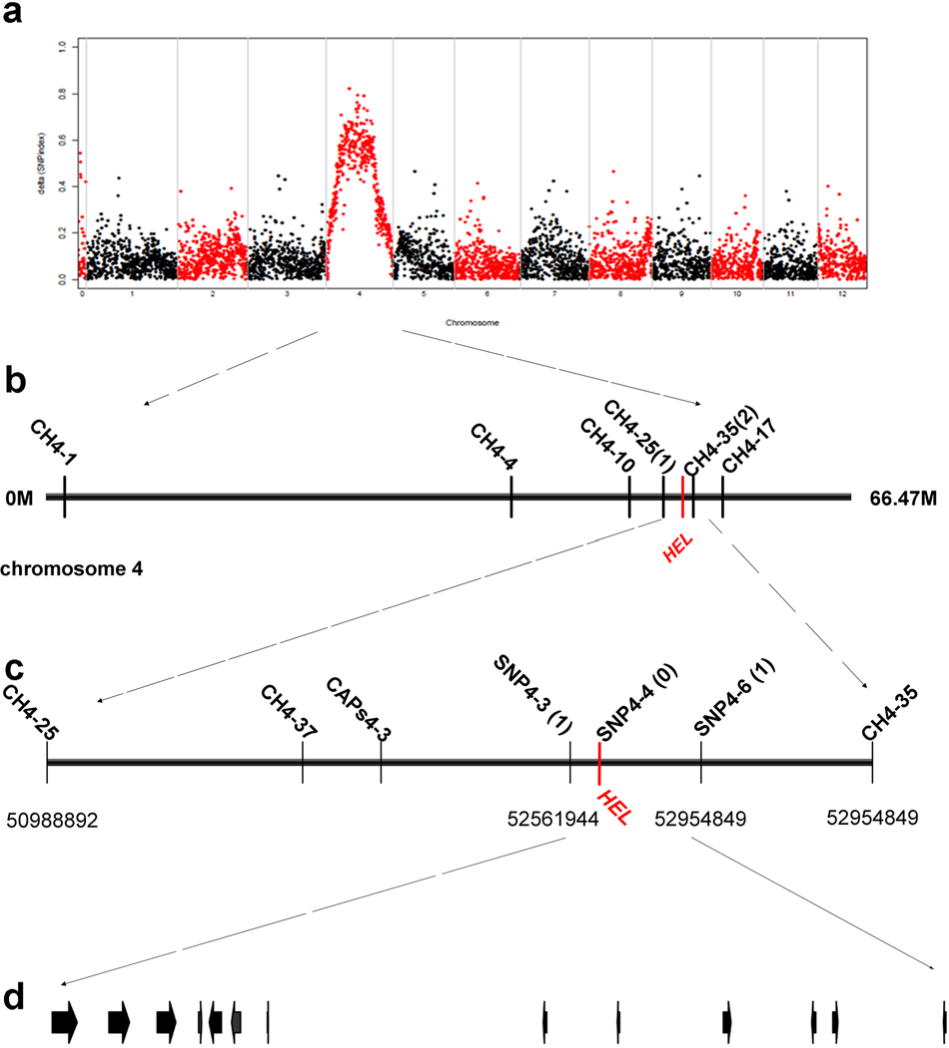
Positional cloning of *HEL*. **a** SNP index graph. The SNP index was calculated based on a 1 Mb interval with a 10 kb sliding window. X-axis, the position of twelve chromosomes; Y-axis, the SNP index. **b** The *HEL* gene was located in the interval between CH4-25 and CH4-35 on chromosome 4. **c** *HEL* was delimited to the region between SNP4-3 and SNP4-4, the physical distance of which was approximately 390 kb. The numbers under the chromosome indicate the physical location based on the tomato genome annotation (ITAG 2.4). **d** Genome annotation demonstrated that this region contains 13 putative ORFs

To determine the chromosomal location of the *HEL* gene, the BSR-seq method was performed using RNA-seq data from the N-pool and M-pool. 7.08 Gb of raw data were generated at an approximately 10× depth and with more than 99% coverage. After removing low-quality bases, 18,517,575 and 22,441,025 clean reads were obtained from the N-pool and M-pool, respectively. The average SNP index of SNPs located within a given genomic interval was then computed. According to the SNP index graph, the peak on chromosome 4 was significantly higher than the other peaks at the 95% significance level, suggesting that this locus may be responsible for the target phenotype (Fig. 3a). To further narrow the interval spanning *HEL*, we developed 6 molecular markers that were evenly distributed along Chr. 4 (Fig. 3b, Supplementary Table S1). Genotyping of 108 F_2_ recessive individuals was performed with these polymorphic markers, and linkage analysis indicated that *hel* was delimited to the region between CH4–25 and CH4–35 at the terminal end of the long arm of Chr. 4, the loci of which were closely linked to *hel* with one and two recombination events, respectively (Fig. 3b).

We further identified nine recombinant individuals by screening 925 recessive F_2_ individuals that possessed CH4-25 and CH4-35. We then used five molecular markers between CH4-25 and CH4-35: CH4-37, CAPS4-3, SNP4-2, SNP4-4, and SNP4-6 (Fig. 3c, Supplementary Table S1). These nine recombinant individuals were genotyped using these newly developed markers (Supplementary Table S1). Finally, we delimited the *HEL* gene to the region between SNP4-3 and SNP4-6, both of which were closely linked to the target gene with just one recombinant (Fig. 3c). SNP4-4 cosegregated with the *HEL* gene (Fig. 3c). Based on the tomato reference genome sequence, the physical distance between SNP4-3 and SNP4-4 was approximately 390 kb (Fig. 3c).

### Candidate genes of *HEL*

Based on the tomato genome annotation (ITAG Release 4.0), the region between SNP4-2 and SNP4-6 encompassed 13 putative ORFs. (Fig. 3d, Table 1). Similar results were also revealed by analysis of the ~390 kb sequence with FGENESH and GeneScan. The best hits of these ORFs include a myosin class II heavy chain protein, a C2 domain-containing protein-like protein, an aspartyl protease family protein, a metal ion-binding protein, a DNA-directed RNA polymerase I subunit RPA12 protein, a peptidyl-prolyl cis-trans isomerase protein, a ring finger protein, a laccase protein, etc.

**Table 1:** Candidates in the region between SNP4-3 and SNP4-6

ORF No.	Accession No.	Location(s)	Gene annotation	Length
1	Solyc04g054470	SL4.0ch04:51856797..51868225	Myosin class II heavy chain	11,427
2	Solyc04g054480	SL4.0ch04:51882810..51892235	C2 domain-containing protein-like	9425
3	Solyc04g054490	SL4.0ch04:51904985..51913563	Aspartyl protease family protein	8620
4	Solyc04g054500	SL4.0ch04:51923982..51925276	Metal ion-binding protein	1295
5	Solyc04g054510	SL4.0ch04:51934475..51928795	DNA-directed RNA polymerase I	5681
6	Solyc04g054520	SL4.0ch04:51943221..51939111	Peptidyl-prolyl cis-trans isomerase	4111
7	Solyc04g054530	SL4.0ch04:51955493..51955972	Mutator-like transposase	480
8	Solyc04g054570	SL4.0ch04:52083688..52082085	Unknown protein	1603
9	Solyc04g054600	SL4.0ch04:52117097..52116177	Unknown protein	920
10	Solyc04g054620	SL4.0ch04:52164740..52168382	Mutator-like transposase-like	3643
11	Solyc04g054630	SL4.0ch04:52207206..52205391	Unknown protein	1816
12	Solyc04g054650	SL4.0ch04:52215059..52217431	Ring finger protein 157	2383
13	Solyc04g054660	SL4.0ch04:52266943..52265856	Laccase	1084

**Fig. 4:**
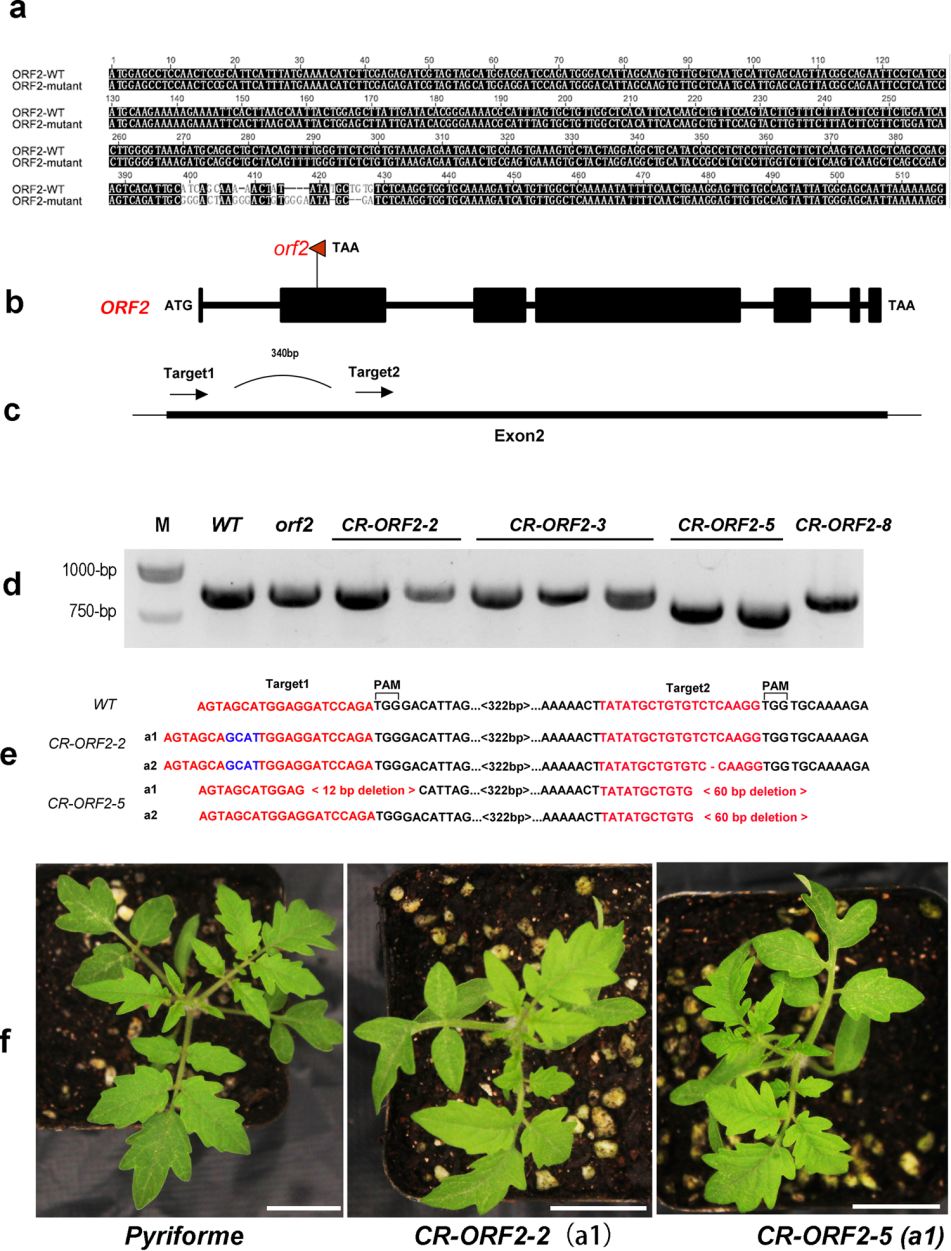
Sequence comparison of two alleles at the *HEL* locus and verification of transgenic ORF2 via the CRISPR-Cas9 system. **a** A 27 bp fragment replacement was detected between the *hel* mutant (ORF2-mutant) and wild-type *Pyriforme* (ORF2-WT). **b** Schematic illustration showing the premature stop codon in the second exon (the red triangle) of *HEL* in the *hel* mutant. **c** Two sgRNAs (arrows) targeting the second exon of ORF2. **d** PCR based identification of 4 CR-*ORF2* T1 lines with a helical growth phenotype. M, marker; WT, wild type; hel, *hel* mutant; *CR-ORF2-2* includes two alleles; *CR-ORF2-5* includes two alleles, with a large fragment deletion. **e** Targeted mutations in CR-*ORF2* transgenic plants. **f** Phenotypes of two *ORF2* edited plants (*CR-ORF2-2*a1 and *CR-ORF2-5*a1). Scale bar, 2 cm

Among these ORFs, ORF2 encodes a C2 domain-containing protein-like protein, the homolog of which is essential for subtle right-handed torsion in *Arabidopsis* (Supplementary Fig. S2)^21^. In addition, ORF13 encodes a laccase protein, and mutation in its *Arabidopsis* homolog *SKU5* gene caused a counterclockwise axial rotation bias^29^. ORF2 and ORF13 were thus considered possible candidates for *HEL*. We amplified and sequenced the full-length coding sequences and the putative promoter fragments of these two candidates from the *hel* mutant and its background material and identified a 27 bp fragment replacement in the *hel* mutant (Fig. 4a). The fragment replacement resulted in a premature stop codon in ORF2 (Fig. 4b). Another candidate showed no sequence changes. These findings further supported the inference that ORF2 was the possible candidate responsible for the helical growth phenotype.

### The *hel* phenotype was complemented by *ORF2* from *Pyriforme*

**Fig. 5:**
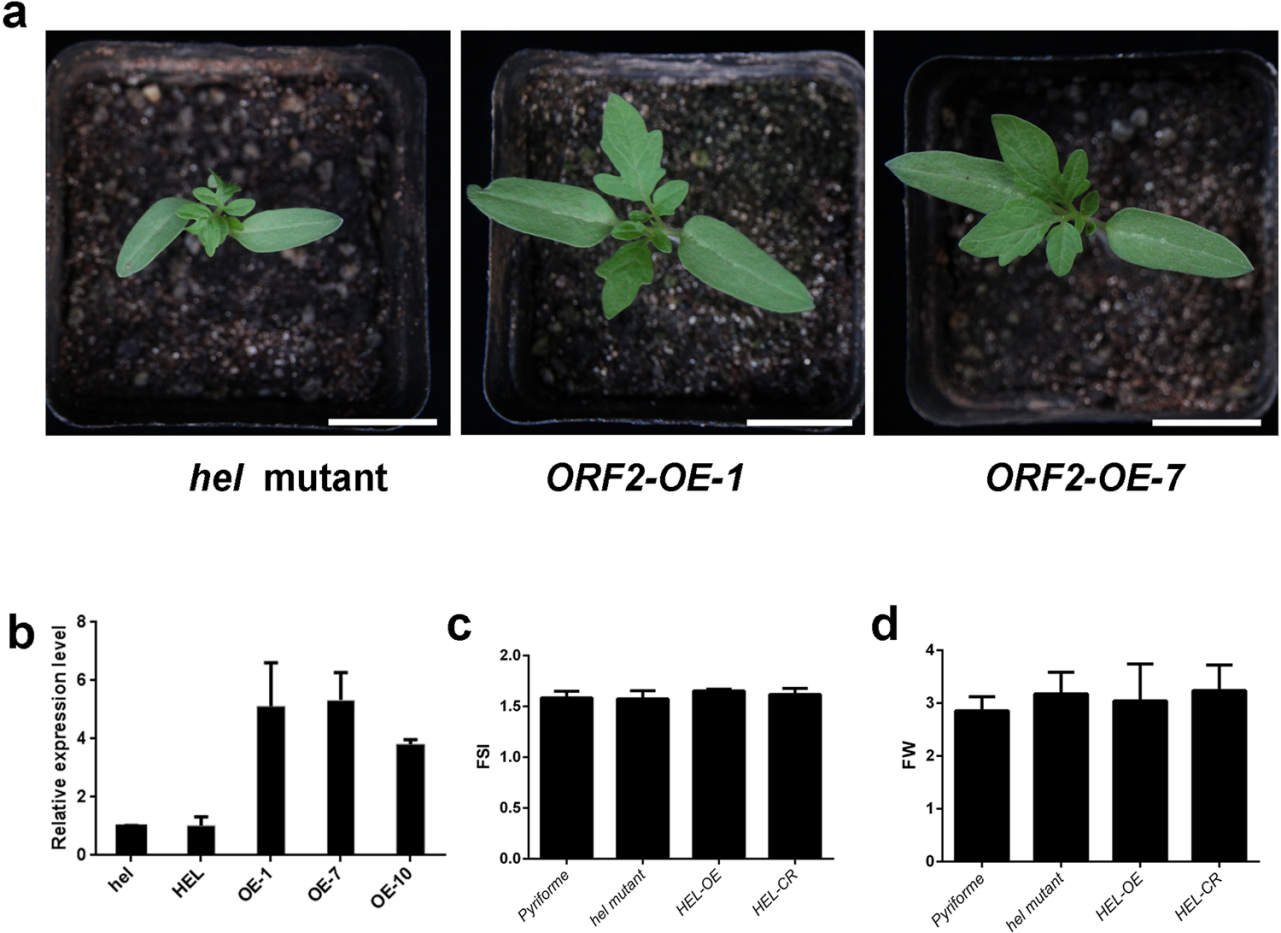
Phenotypes of ORF2 overexpression lines. **a** The phenotype of the *hel* mutant was restored by ORF2 overexpression. **b** Expression level of ORF2 in the *hel* mutant, *Pyriforme* and overexpression lines. **c** and d The fruit shape index (FSI) and fruit weight (FW) of *Pyriforme*, the *hel* mutant, and ORF2 overexpression lines. Scale bar, 2 cm

To support that ORF2 is responsible for the target phenotype, we prepared a CRISPR/Cas9 vector and transformed this vector into *Pyriforme* via Agrobacterium-mediated transformation. Two adjacent target sites were selected in the second exon of ORF2 (Fig. 4c). Editing of ORF2 in the CRISPR lines was detected via PCR and Sanger sequencing (Fig. 4d, e). The CRISPR line *CR-ORF2-2* contained a 4 bp insertion in the first target. The CRISPR line *CR-ORF2-5a1* contained a 12 bp deletion downstream of the first target and a 60 bp deletion downstream of the second target (Fig. 4d, e). These mutations in ORF2 resulted in a helical growth phenotype similar to that of the *hel* mutant (Fig. 4f). To further examine the biological function of ORF2, an overexpression construct for this candidate gene under the control of the CaMV-35S promoter was prepared and introduced into the *hel* mutant. Eight independent transformants were obtained. The helical growth phenotype of the *hel* mutant was complemented by the fragment from *Pyriforme* (Fig. 5a). QRT-PCR analysis indicated that the expression level of ORF2 obviously increased in the transgenic plants (Fig. 5b). Taken together, these results suggest that ORF2 is the right candidate for *HEL*. Notably, the fruits of the transgenic plants showed no obvious differences compared to those of the *hel* mutant and *Pyriforme* (Fig. 5c, d).

## Discussion

### BSR-seq combined with molecular marker analysis is an efficient method for gene cloning

Bulked segregant analysis (BSA) has been extensively applied to identify molecular markers linked to a chromosomal region associated with a target phenotype^30^. Two bulk DNA samples collected from wild-type and mutant individuals separately need to be sequenced via whole-genome shotgun sequencing. Although the sequencing price has sharply dropped with rapid improvement of the technology underlying genome sequencing, this strategy is still not cost-effective in tomato. BSA in combination with RNA-seq was therefore developed as an efficient and cost-effective strategy for determining the chromosomal location of target genes^25,31–33^. After the approximate region of the target gene was determined by the BSR-seq method, fine-mapping with molecular markers has been suggested to be feasible and effective in many crop species, such as wheat and cotton^34–36^. In this study, we first adopted the BSR-seq method to identify the chromosomal location of the *HEL* gene. *HEL* was successfully mapped to chromosome 4 via a low-depth BSR-seq approach. Combining BSR-seq with classic marker analysis, we delimited *HEL* to within the region between markers SNP4-2 and SNP4-6. The efficiency of gene mapping with BSR-seq analysis and marker screening obviously improved.

### *HEL* possibly controls tomato helical growth via molecular mechanisms different from those in *Arabidopsis*

In this study, we determined that *HEL* encodes a cellulose synthase interactive protein in tomato. There are 3 *HEL* homologous genes in *Arabidopsis*: *CSI1*, *CSI2* and *CSI3*. HEL shared 79% amino acid sequence identity with CSI1, 38.5% with CSI2 and 44.5% with CSI3 (Fig. S2). CSI1 acts as a physical link between CESA complexes and cortical microtubules^19,20,37^. Previous investigations suggested that CSI1, CSI2, and CSI3 exhibited similar functions in guiding primary CSCs along CMTs during cellulose biosynthesis^38^. The orientation of cellulose microfibrils and cortical microtubules in mutants with mutations in the *CSI1* gene was uncoupled^19,20^. Furthermore, elongating roots, hypocotyls, and leaves of the *csi1* mutant showed left-handed spiraling, whereas the inflorescence stems exhibited slightly right-handed spiraling twists^20,21^. Here, we found that the stems of the *hel* mutant exhibited left-handed twisting and vine-like growth after flowering; nevertheless, the leaves that initially showed right-handed helical growth became left-handed as the plants grew. However, similar phenotypes were not observed for the *Arabidopsis csi1* mutant. Therefore, it can be speculated that the molecular mechanism mediated by *HEL* underlying helical growth of tomato is different from that of *Arabidopsis*. In tomato, the *corkscrew* (*crs*) mutant also exhibited helical growth^39^. However, the phenotypic details of cotyledons, leaves, stems, and anther cones of the *crs* mutant differed from those of the *hel* mutant. It would be worthwhile to clone the *CRS* gene and determine the possible roles of this gene in helical growth of tomato.

### HEL may work together with MAPs during helical growth formation

The microtubule–microfibril alignment model laid a theoretical foundation for the helical growth model. It is still not clear why mutations in highly conserved proteins such as HEL and CSI1 cause opposite phenotypes. The different directions of helical growth of *Arabidopsis* are caused by a series of mutations in tubulins or MAPs^5–10,29,40,41^. CSI1 plays an important role in sustaining CSC movement along CMTs. Moreover, the movement direction is dependent on other MAPs, such as kinesins^40^. As expected, mutations in kinesin resulted in right-handed helical root growth^42^. Hence, it can be deduced that the microtubule–microfibril alignment model is governed by complex variable systems that can be mediated by multiple protein complexes. These different combinations of proteins could lead to different phenotypes. In this study, the fruits of *Pyriforme* exhibited a pear shape, similar to those of the *ovate* mutant. OVATE has been proven to play an essential role in microtubule arrangement by interacting with TON1 Recruiting Motif (TRM) proteins^43,44^. Moreover, the stems of the *hel* mutant showed no helical growth at the early stage, which gradually shifted to left-handed twisting after flowering. This shift was closely related to the expression level of *OVATE*^45^. We thus infer that these MAPs may be involved in the regulation of tomato helical growth by interacting with HEL.

### Application of CRISPR/Cas9 efficiently restructured the erect architecture of tomato to a helical architecture

Recently, the CRISPR/Cas9 strategy has been successfully used to reprogram the vine-like structure of plants into a compact stature^46,47^. Both HEL and CSI1 are essential for helical growth of tomato and *Arabidopsis*, suggesting that members of this family can be edited to alter the growth habit of other plant species. In the present study, we mutagenized *HEL* using the CRISPR/Cas9 system and obtained several mutant lines. Compared with those of erect tomato plants, the stems of the mutated lines showed a left-handed helix and twined around a support under artificial traction, which can help save time required for winding onto a support and the amount of ties and ropes. The inflorescence branches and fruits of the *hel* mutant showed no obvious differences compared to those of wild-type plants. Although the tomato fruit set of the *hel* mutant decreased, elucidating the molecular basis underlying tomato helical growth may provide a new idea for formulating modern sustainable agricultural practices in the future.

## Supplementary information

Phenotype of hel mutant and Pyriforme

S2 Alignment between HEL and CSIs in Arabidopsis

The information of the markers for mapping

Genotyping of the immortal critical recombinants (ICRs)
