# Supplementary figures and images for: The loss of function of HEL, which encodes a cellulose synthase interactive protein, causes helical and vine-like growth of tomato
Source: Hortic Res. 2020 Nov 1;7:180. doi: 10.1038/s41438-020-00402-0 (PMC7603515; doi:10.1038/s41438-020-00402-0)

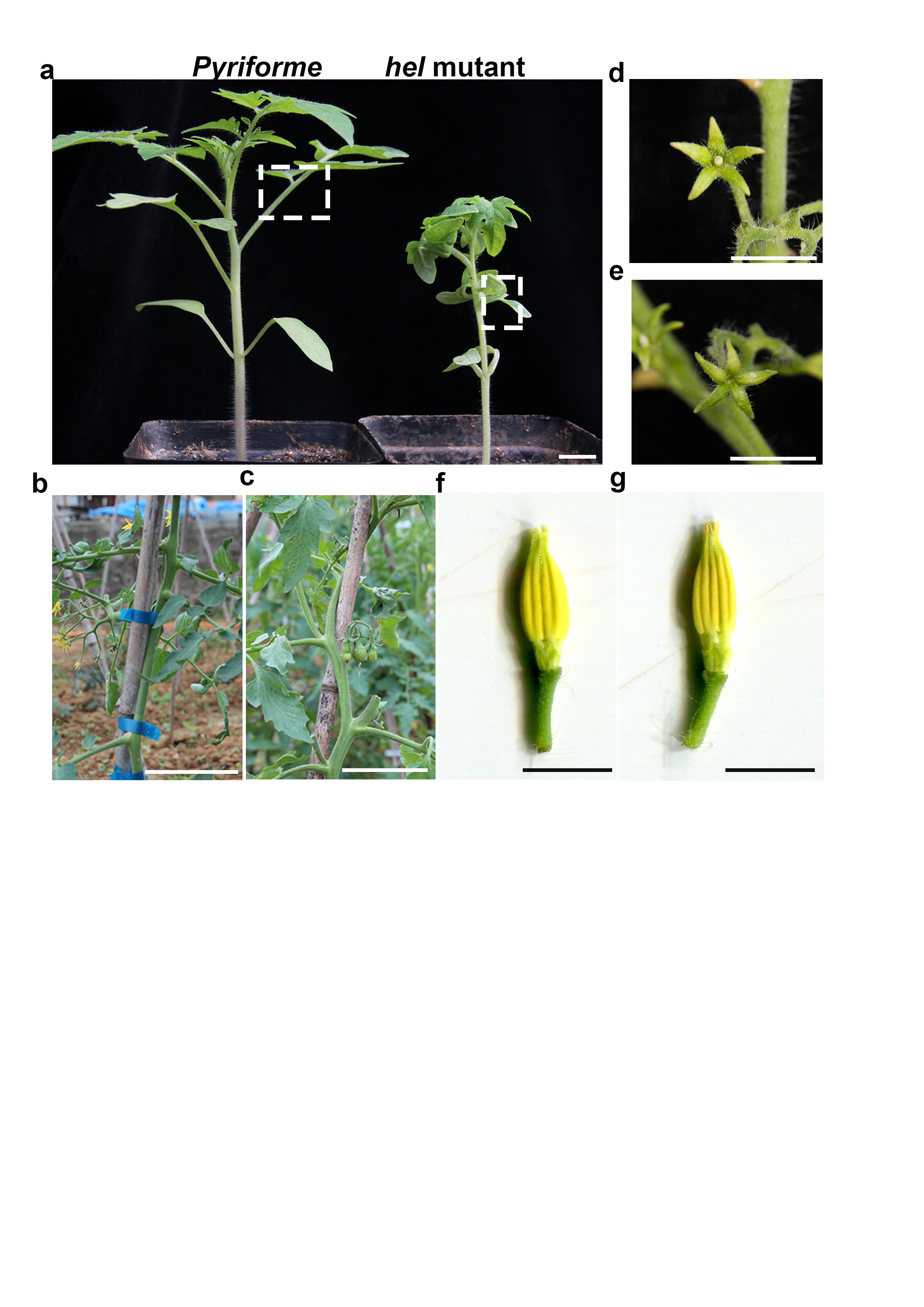

Supplement: Supplementary file 1 — Phenotype of hel mutant and Pyriforme [file 41438_2020_402_MOESM1_ESM.jpg]

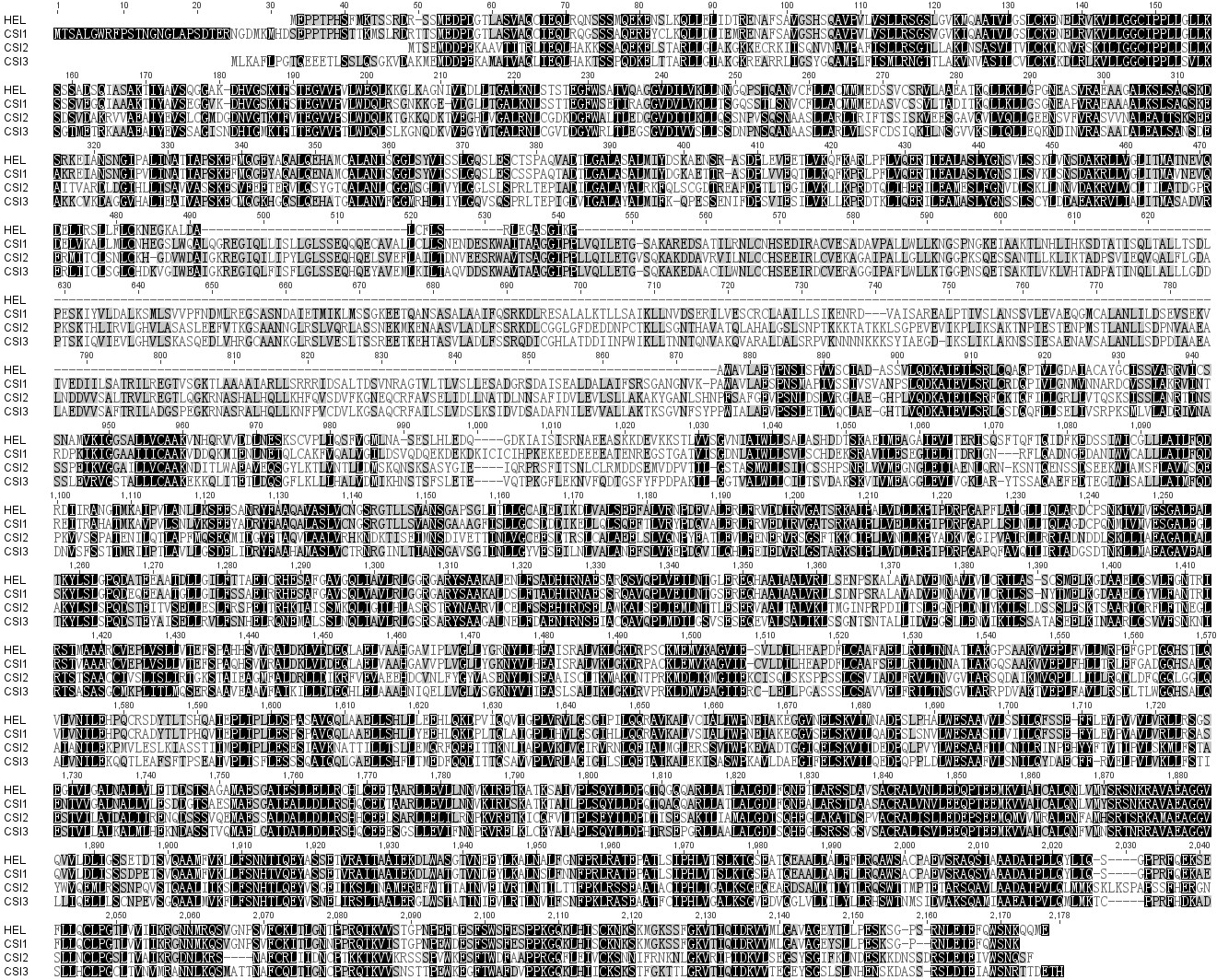

Supplement: Supplementary file 2 — S2 Alignment between HEL and CSIs in Arabidopsis [file 41438_2020_402_MOESM2_ESM.jpg]
