# Supplementary material for: The loss of function of HEL, which encodes a cellulose synthase interactive protein, causes helical and vine-like growth of tomato
Source: Hortic Res. 2020 Nov 1;7:180. doi: 10.1038/s41438-020-00402-0 (PMC7603515; doi:10.1038/s41438-020-00402-0)
Supplement: Supplementary file 3 — The information of the markers for mapping [file 41438_2020_402_MOESM3_ESM.pdf]

Table S1.The information of the markers for mapping

Maker type	Maker name	Location(s)	Forward primer(5'–3')	Reverse primer (5'-3')	PCR product length(bp)	Amplify temp. (℃)	Restriction enzyme
Indel	CH4-1	SL2.40ch04:1533907..1534029	AGACCGTATTTTATGATTGTGGCTCC	ACTCATCAGCAAAGGCAAGGACC	452	55	
Indel	CH4-4	SL2.40ch04:37590200..37590201	CAAGAGATTGATCCTCCAGAAAGA	CACTAACTTTTACATGGAACAACCC	563	54	
Indel	CH4-10	SL2.40ch04:47339923..47339924	GTTGGCTTATTTAGGCATTTCATCG	TGTTTCCGTCTCATTCTGCTTCT	325	55	
Indel	CH4-17	SL2.40ch04:55006570..55006694	GCTGAGAAGAAGAAGAAACCAAAGA	TTGTGATAATGGGAGGTCATGTTC	548	55	
Indel	CH4-25	SL2.40ch04:50136361..50136362	GACGATGCACATGCAGGTTAGTT	ATGAAAAGTGGTTAGGTGGTAGGAA	327	55	
Indel	CH4-35	SL2.40ch04:52615710..52615715	CTCAATCTTGTTCATATCCACCCT	TAGTAGAGCCTATCATTGAAACACCT	280	55	
Indel	CH4-37	SL2.40ch04:50905127..50905171	GGATCACATGAGAAAGGCAAGCT	TAACCACGAATGTACTGCAACCC	440	55	
Caps	CAPS4-3	SL2.40ch04:51286129..51286230	GGAGGAAAGCAGAAAGAGGAAAC	TCTACCAGCAATCATACCCAACC	1273	56	DraI
SNP	SNP4-2	SL2.40ch04:51708962..51709010	AACAAACCCACGAGTAAGAAGAC	AAATGAGACGGAGGAAGTAACAG	885	54	
SNP	SNP4-4	SL2.40ch04:52740741..52741440	ACATACCATCCCATTAGACTCCC	CCTCATCCTCCTATCGCTACATC	700	54	
SNP	SNP4-6	SL2.40ch04:52102439..52102500	TGGAGCCTATGACTGGAGATTTA	GATACCTTCAGGTGTTCGGATTA	737	54	
