# Supplementary material for: The loss of function of HEL, which encodes a cellulose synthase interactive protein, causes helical and vine-like growth of tomato
Source: Hortic Res. 2020 Nov 1;7:180. doi: 10.1038/s41438-020-00402-0 (PMC7603515; doi:10.1038/s41438-020-00402-0)
Supplement: Supplementary file 4 — Genotyping of the immortal critical recombinants (ICRs) [file 41438_2020_402_MOESM4_ESM.pdf]

Table S2. Genotyping of the immortal critical recombinants (ICRs).

Maker	hel mutant	LA1589	F1	F2 Individuals With Helical Growth Phenotype															
				5	14	16	20	33	54	57	62	68	91	94	205	243	272	304	313
CH4-1	a	b	h	h	h	a	h	a	h	a	a	h	a	a	h	h	a	a	h
CH4-4	a	b	h	h	h	a	h	a	h	a	a	h	a	a	h	h	a	a	h
CH4-10	a	b	h	h	h	a	a	a	h	a	a	h	a	a	h	h	a	a	h
CH4-25	a	b	h	a	h	a	a	a	h	a	a	h	a	a	h	h	a	a	h
CH4-37	a	b	h	a	a	a	a	a	h	a	a	h	a	a	h	h	a	a	h
CAPS4-3	a	b	h	a	a	a	a	a	h	a	a	a	a	a	h	a	a	a	a
SNP4-2	a	b	h	a	a	a	a	a	a	a	a	a	a	a	h	a	a	a	a
SNP4-4	a	b	h	a	a	a	a	a	a	a	a	a	a	a	a	a	a	a	a
SNP4-6	a	b	h	a	a	a	a	a	a	h	a	a	a	a	a	a	a	a	a
CH4-35	a	b	h	a	a	a	a	a	a	h	h	a	h	a	a	a	h	h	a
CH4-17	a	b	h	a	a	h	a	h	a	h	h	a	h	h	a	a	h	h	a

Note: The "a" represents genotype of female parent, the "b" represents genotype of male parent, the "h" represents hybird genotype
